# Intertrochanteric fractures in elderly high risk patients treated with Ender nails and compression screw

**DOI:** 10.4103/0019-5413.65154

**Published:** 2010

**Authors:** Sidhartha Gangadharan, MR Nambiar

**Affiliations:** Department of Orthopaedics, Satchidananda Institute of Medical Sciences, Anandashram, Kanhangad, Kerala - 671 531, India

**Keywords:** Compression screw, Ender nails, osteoporosis, inter-trochanteric fracture

## Abstract

**Background:**

Ender and Simon Weidner popularized the concept of closed condylocephlic nailing for intertrochanteric fractures in 1970. The clinical experience of authors revealed that Ender nailing alone cannot provide secure fixation in elderly patients with osteoporosis. Hence we conducted a study to evaluate the efficacy of a combined fixation procedure using Ender nails and a cannulated compression screw for intertrochanteric fractures.

**Materials and Methods:**

76 patients with intertrochanteric fractures were treated using intramedullary Ender nails and cannulated compression screw from January 2004 to December 2007. The mean age of the patients was 80 years (range 70-105 years).Using the Evan’s system of classification 49 were stable and 27 unstable fractures. Inclusion criteria was high risk elderly patients (age > 70 years) with intertrochanteric fracture. The exclusion criteria included patients with pressure sores over the trochanteric region. Many patients had pre-existing co-morbidities like diabetes mellitus, hypertension, COPD, ischemic heart disease, CVA and coronary artery bypass surgery. The two Ender nails of 4.5mm each were passed across the fracture site into the proximal neck. This was reinforced with a 6.5 mm cannulated compression screw passed from the sub trochanteric region, across the fracture into the head.

**Results:**

The mean follow-up was 14 months (range 9-19 months) Average time to fracture union was 10 weeks (range 6-16 weeks). The mean knee ROM was 130° (± 5°). There was no case of nail penetration into hip joint. In five cases with advanced osteoporosis there was minimal migration of Ender nails distally.

**Conclusions:**

The Ender nailing combined with compression screw fixation in cases of intertrochanteric fractures in high risk elderly patients could achieve reliable fracture stability with minimal complications.

## INTRODUCTION

The Ender nail for intramedullary fixation of intertrochanteric fractures of the femur^1^ was developed by Ender and Simon Weidner and further simplified by Kuntscher. The tensile property of the nail combined with the simplicity of the procedure and feasibility of early ambulation led to the nail becoming popular among hip surgeons. However, the nail ran out of favor on account of its failure to control rotation and distal migration in previous series.^1^ Bearing this in mind, we added a cannulated compression screw to the Ender nails in an attempt to maintain fracture reduction. Hence a study regarding the feasibility of a combined procedure in high risk elderly cases was conducted.

## MATERIALS AND METHODS

**Figure 1:**
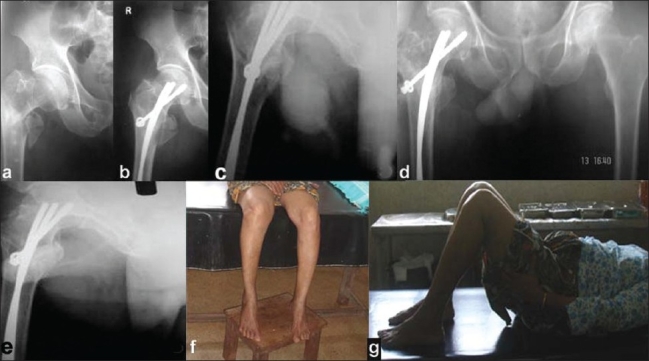
(a) Pre-operative X-ray of right hip joint of a 74-year-old female with unstable intertrochanteric fracture six weeks. Post-operative antero-posterior (b) and lateral (c) radiographs of right hip showing satisfactorily maintained fracture reduction and implant in situ. Ten months follow-up antero-posterior (d) and lateral (e) radiographs of right hip showing fracture union with good alignment and clinical photographs (f, g) showing knee and hip range of motion.

This prospective study includes 76 intertrochanteric fractures without subtrochanteric extension in high risk elderly cases presented between Jan 2004 and Dec 2007. There were 49 stable and 27 unstable intertrochanteric fractures (as per Evan’s classification) [Figure 1a]. We included elderly patients of age more than 70 years having sustained a closed fracture without any pressure sores. The presence of co-morbidities like diabetes mellitus (n=33), hypertension (n=29), COPD (n=11), ischemic heart disease (n=8), CVA (n=2) and history of previous coronary artery bypass surgery (n=1) were also included. The mean age of the patients was 80 years (range 70-105 years); 27 were males and 49 were females. All patients were ambulant before sustaining fractures, except two hemiparetic patients who needed support to walk. All patients were treated within four days of the fracture. The clinical and radiolographical assessment was done in all cases. A detailed informed consent was taken from each patient.

The mean follow-up is 14 months (range 9-19 months) [Figure 1b and c].

### Operative procedure

Fracture reduction was attained under image intensifier over a fracture table. The appropriate nail size was ascertained by trial placements of nail over the limb using an image intensifier. This was followed by insertion of two Ender nails (4.5 mm diameter), 1cm proximal to the adductor tubercle into the canal. The nails were then advanced across the fracture site into the proximal neck. After drilling and tapping a single 6.5 mm cannulated compression screw was introduced across the fracture from the subtrochanteric region into the femoral head. The distal migration of Enders nail was prevented by passing a K-wire through the eye of the nail in all cases using image intensifier.

Quadricep strengthening exercises were encouraged from the first postoperative day. Non-weight bearing ambulation touch toe using a walker was permitted in self confident patients by the 10^th^ post-operative day. Patients were called for review after a month and assessed clinically for any limb length discrepancy and mal alignment of the limb. Radiological assessment was done to verify the position of the implant as a check to compliance with the postoperative ambulation protocol. During the first followup at one month xray pelvis with both hips anteroposterior (AP) view and involved hip lateral was done. Partial weight bearing was initiated after the sixth week. It was gradually progressed to full weight bearing as per tolerance and absence of radiological evidence of collapse [Figure 1d and e]. Successive reviews were done at six-week intervals during which rotations in flexion/extension, limb length discrepancy and knee range of motion were assessed [Figure 1f and g]. In the event of patient complaining knee pain, X-ray distal femur with knee AP was done.

## RESULTS

The average blood loss during the surgery was 20 cc (range 15 to 40 cc). The average surgery time was 35 minutes (range 25 to 45 minutes). The average time taken for fracture union was 10 weeks (range 6 to 16 weeks). None of the fractures united in varus. There were no cases of the compression screw backing out or the nail cutting out proximally. Ender nails in five cases of advanced osteoporosis migrated distally though there weren’t any case of penetration into the knee joint as the entry point was from the adductor tubercle. Patients in those cases complained of stiffness and pain above the knee due to painful bursa formation. There was mild external rotation deformity of 10° in four cases. Limb shortening (1 to 2 cm) resulted from the collapse of the comminuted fragments in six cases. There were no other complications such as DVT, wound infection or non union. Using the modified Harris hip score results were fair bearing in mind the mean age of the series being 80 years and oldest being 105 years. The pre-operative and the post-operative mean modified Harris hip scores were 63 and 72 respectively with an appreciable change in score of 9.

## DISCUSSION

Many types of internal fixation devices have been introduced for intertrochanteric fracture. Any surgical treatment with fixation devices for this fracture should provide sufficient fixation of the fracture to allow early mobilization of the fractured limb, to obtain fracture union, and to minimize the complications such as delayed union or nonunion, penetration of the nail into the hip joint and distal migration. In patients with osteoporosis, any single type of internal fixation device cannot provide secure fixation of the fracture, resulting in loss of the reduced position together with migration of the nails.^2^ Presently, intertrochanteric fractures are fixed either with dynamic hip screw or proximal femoral nail.^3^ Both these methods though providing secure fixation have their drawbacks. Dynamic hip screw (DHS) is complicated by joint penetration and cut out in osteoporotic patients.^4^ Both these complications are catastrophic for the patient and surgeon. DHS also entails significant blood loss and traumatic in high risk cases.^5^

Proximal femoral nail (PFN) is technically demanding and dependent on the status of pyriform fossa. In a patient with fracture involving pyriform fossa, PFN is not ideal. PFN also carries an unacceptably high risk of fracture of femur at the tip of the nail.^5^ Ender nails alone have also been used in fixation of intertrochanteric fractures.^6^,^14^ Past authors reported an unacceptably high failure rate with Ender nails alone.^7^,^15^,^16^ The Ender nail used alone did not provide rotational stability and was associated with an increased risk of migration and joint penetration proximally or distally.^8^,^13^

There are no reports of the combined procedure of Ender nailing and compression screw to best of our knowledge for intertrochanteric fracture in English language literature^9^–^12^. By incorporating the tensile property of Ender nails along with a compression screw, fracture reduction and prevention of rotation respectively were possible. This combination tended to augment the fracture stability in presence of osteoporosis.^17^ However, the combined procedure brought successful union in all cases which could be listed as a merit. In none of this series did the nail tips penetrate or cut the head. The comparative common postoperative complaints were pain around the knee joint and minimal residual stiffness of the knee. Combination fixation of intertrochanteric fractures with Ender nails and compression screw is technically less demanding, minimally invasive, entails less operative time (beneficial factor in high risk cases) and least traumatic with minimal blood loss. This method can be used irrespective of the status of pyriformis fossa and has proved to be an ideal alternative procedure for fixation of intertrochanteric fractures in elderly patients with high risk co-morbidities and osteoporosis, which can be carried our in an average orthopedic setup.

The Ender nailing combined with compression screw fixation in cases of inter intertrochanteric fractures in high risk elderly could achieve reliable fracture stability with minimal complications.
